# A randomised trial of a psychosocial intervention for cancer patients integrated into routine care: the PROMPT study (promoting optimal outcomes in mood through tailored psychosocial therapies)

**DOI:** 10.1186/1471-2407-11-48

**Published:** 2011-02-01

**Authors:** Jane Turner, Brian Kelly, David Clarke, Patsy Yates, Sanchia Aranda, Damien Jolley, Suzanne Chambers, Maryanne Hargraves, Lisa McFadyen

**Affiliations:** 1 School of Medicine, University of Queensland and Royal Brisbane and Women's Hospital, Brisbane, Australia; 2 University of Newcastle and John Hunter Hospital, Newcastle, Australia; 3 Monash Medical Centre, Melbourne, Australia; 4 Queensland University of Technology, Brisbane, Australia; 5 University of Melbourne and Peter MacCallum Cancer Centre, Melbourne, Australia; 6 School of Public Health and Preventive Medicine, Monash University, Melbourne, Australia; 7 Cancer Council Queensland, Brisbane, Australia; 8 Haematology and Oncology Clinics of Australasia, Brisbane, Australia; 9 Melanoma Patients Australia, Brisbane, Australia

## Abstract

**Background:**

Despite evidence that up to 35% of patients with cancer experience significant distress, access to effective psychosocial care is limited by lack of systematic approaches to assessment, a paucity of psychosocial services, and patient reluctance to accept treatment either because of perceived stigma or difficulties with access to specialist psycho-oncology services due to isolation or disease burden. This paper presents an overview of a randomised study to evaluate the effectiveness of a brief tailored psychosocial Intervention delivered by health professionals in cancer care who undergo focused training and participate in clinical supervision.

**Methods/design:**

Health professionals from the disciplines of nursing, occupational therapy, speech pathology, dietetics, physiotherapy or radiation therapy will participate in training to deliver the psychosocial Intervention focusing on core concepts of supportive-expressive, cognitive and dignity-conserving care. Health professional training will consist of completion of a self-directed manual and participation in a skills development session. Participating health professionals will be supported through structured clinical supervision whilst delivering the Intervention. In the stepped wedge design each of the 5 participating clinical sites will be allocated in random order from Control condition to Training then delivery of the Intervention. A total of 600 patients will be recruited across all sites. Based on level of distress or risk factors eligible patients will receive up to 4 sessions, each of up to 30 minutes in length, delivered face-to-face or by telephone. Participants will be assessed at baseline and 10-week follow-up. Patient outcome measures include anxiety and depression, quality of life, unmet psychological and supportive care needs. Health professional measures include psychological morbidity, stress and burnout. Process evaluation will be conducted to assess perceptions of participation in the study and the factors that may promote translation of learning into practice.

**Discussion:**

This study will provide important information about the effectiveness of a brief tailored psychological Intervention for patients with cancer and the potential to prevent development of significant distress in patients considered at risk. It will yield data about the feasibility of this model of care in routine clinical practice and identify enablers and barriers to its systematic implementation in cancer settings.

**Trial registration:**

ACTRN12610000448044

## Background

### The mental health burden of cancer

Approximately 35% of patients with cancer experience clinically significant distress, patients with poorer prognosis and those with higher levels of disease burden being especially at risk [1]. The International Psycho-Oncology Society (IPOS) is the international multidisciplinary body which advocates for attention to psychological, social and behavioural issues in cancer care. In 2010 IPOS proposed a new international quality standard, endorsing Distress as the "Sixth Vital Sign" in order to raise awareness of the need to evaluate and monitor distress as part of routine cancer care. However strategies to embed such evaluation and subsequent interventions into routine care have received less attention. In Australia Clinical Practice Guidelines for the Psychosocial Care of Adults with Cancer (PCPG) describe the benefits of psychosocial Interventions in this population to promote well-being [2], and there is emerging evidence that psychosocial Interventions may prevent the development of frank disorder such as Major Depression [3]. Despite this evidence, rates of referral for specialist treatment are low - in one Australian study 75% of patients with clinically significant anxiety or depression did not receive any counselling or psychological treatment [4]. Low referral rates may reflect limited clinician ability to detect distress, exemplified by research in which oncologists identified only 17% of clinically anxious and 6% of clinically depressed patients [5]. This limited detection may relate to patient reluctance to volunteer emotional concerns because of the belief that distress is inevitable, or that nothing can be done to assist [6] meaning that health professionals must often rely on non-verbal cues to identify distress, a difficult task in the absence of specific training [7]. Stigma about mental illness may be another reason that patients are reluctant to disclose emotional concerns or accept referral if this is offered [8,9].

Limited access to suitably qualified professionals to provide psychosocial care may undermine clinician enthusiasm about detecting distress - why identify a problem if it cannot be addressed? But even in settings with access to specialist staff, psychosocial care is not uniformly offered. For example a cross-sectional survey found that psychosocial advice was provided for up to 50% of patients at diagnosis, but at completion of treatment patients were seldom given advice about adjustment to survivorship [10], despite the fact that the transition from active treatment to follow-up is a time of increased psychological vulnerability [2].

In addition to the obvious workforce barriers to provision of optimal psychosocial care, broader systems issues are likely to play a role. In clinical services under severe pressure to provide patient care despite limited resources, psychosocial care may be assigned a lower priority than biomedical care. In these settings, absence of defined pathways to routinely assess and address psychosocial need means that these issues "slip under the radar", with the consequence that patient need is unmet. Depression and anxiety have a profound adverse impact on well-being of patients with medical illness [11] and thus merit treatment in their own right. However at a systems level, it is vital to recognise that depression and anxiety are associated with reduced capacity to cope with disease burden, more frequent presentations to health services and increased costs [12], putting further strain on a struggling health system.

### Addressing barriers to care - implementation of systematic flexible approaches embedded in clinical care

Given the limitations of health professionals' identification of patient distress, implementation of systematic evidence-based approaches to screening and detection of distress is essential to ensure that patients' needs are appropriately identified [13]. Instruments such as the Distress Thermometer which has an accompanying problem list provide a starting point for assessment of each patient's unique needs [14]. Patient need may also relate to vulnerability, identified by the presence of risk factors, even in the absence of current distress. The Australian National Breast and Ovarian Cancer Centre (NBOCC) has developed a structured template to promote a systematic approach to risk assessment [15], based on evidence derived from the Clinical Practice Guidelines for the Psychosocial Care of Adults with Cancer [2]. However to date there has been no research examining psychosocial Interventions in this vulnerable population, in particular whether provision of information and self-management strategies might *prevent *the development of disorder such as depression.

Having identified patient distress, a critical next step is to determine the severity and sources of distress. International research highlights the need to move beyond generic ("one size fits all") approaches to an *individualised *approach, for example addressing issues such as dignity and personal integrity [16]. This is consistent with a tiered model of care in which psychosocial interventions are tailored to the individual's level of need [17] - only the most distressed patients are offered specialist referral - the needs of patients with mild distress being addressed through more basic supportive interventions, often delivered by non-specialist health professionals. Our research has demonstrated that focused training leads to improved knowledge, confidence and skills of oncology nurses [18,19] meaning that nurses could potentially deliver psychosocial care for less severely distressed patients. A UK study supports the feasibility of this model of care, demonstrating that oncology nurses without prior training in psychosocial interventions can be trained to deliver a brief Intervention for depressed cancer patients, leading to improvement in symptoms of depression [20]. In addition to the obvious implications in terms of improving patient access to care, such an approach is likely to counter stigma as support provided by breast care nurses is regarded by patients as both effective and highly acceptable [21]. A further advantage of an approach which is embedded in routine clinical care is that patients who have high disease morbidity may find this support more accessible and timely.

Another strategy to enhance acceptability to patients and improve sustainability is to adopt more flexible approaches to the delivery and style of interventions. For example telephone-based counselling is likely to be highly acceptable to patients from rural areas or those with disease burden which limits travel. Randomised trials of telephone-based interventions have been successful in improving patient pain and depression [22] and distress [23]. Adaptation of traditional time-intensive psychosocial therapies into brief, more flexible formats is likely to improve acceptability, and also increases the feasibility of these interventions being incorporated into routine clinical care. This approach is supported by evidence: simple behavioural therapies appear to be as effective as more complex cognitive techniques [24], and a component analysis of therapy for treatment of depression concluded that simple behavioural activation was as effective as more complex treatments [25]. Minimal-contact psychotherapy has also been demonstrated to be effective for depressed patients in primary care [26], and brief targeted therapy for medically-ill patients experiencing demoralisation has been described [27].

## Methods/Design

### Study aims and hypotheses

The study's primary aim is to evaluate the effectiveness of a brief psychosocial Intervention in reducing depression and anxiety in patients with cancer who are distressed, but not severely depressed, or who are at risk of becoming depressed. The Intervention will be delivered by health professionals who have participated in focused training and who receive clinical supervision during delivery of the Intervention. Additional aims are to examine the impact of the psychosocial Intervention on patient unmet needs, quality of life, and scores of demoralisation.

Secondary aims are to examine any impact on stress and burnout and psychological adjustment of the health professionals who deliver the psychosocial Intervention, to assess the feasibility of integration of the Intervention into clinical practice, and to identify health professional, systems and patient barriers to the implementation of this model of systematic tailored psychosocial Interventions in clinical care.

It is hypothesised that:

1. Patients receiving the Intervention will demonstrate greater improvement in depression/anxiety scores and reduction in depression/anxiety "caseness" compared with patients receiving Usual Care.

2. The Intervention will be associated with lower rates of depression/anxiety and increasing reduction in depression/anxiety scores over time in patients of the cancer services.

3. There will be no changes in health professional stress and burnout.

### Intervention

This study comprises two discrete components:

a) Training of health professionals who will deliver the psychosocial Intervention

b) Delivery of the psychosocial Intervention by the trained health professionals.

The design for this randomised study is a stepped-wedge cluster design, a type of crossover design in which different clusters (the clinical sites) cross over in one direction only, from Control to Training then Intervention [28]. At the conclusion of the study, all sites will have received Training and be providing the Intervention [29] delivered by health professionals who have received training at that site as depicted in Table 1.

**Table 1:** Stepped wedge design.

	Epoch 1	Epoch 2	Epoch 3	Epoch 4	Epoch 5	Epoch 6	Epoch 7
Site 1	Control	TRAINING	**Intervention**	**Intervention**	**Intervention**	**Intervention**	**Intervention**

Site 2	Control	Control	TRAINING	**Intervention**	**Intervention**	**Intervention**	**Intervention**

Site 3	Control	Control	Control	TRAINING	**Intervention**	**Intervention**	**Intervention**

Site 4	Control	Control	Control	Control	TRAINING	**Intervention**	**Intervention**

Site 5	Control	Control	Control	Control	Control	TRAINING	**Intervention**

Week	1	10	20	30	40	50	60

The Intervention is sequentially rolled-out across sites in random order over a number of time periods (Epochs).

The nature of the Intervention will be *tailored to the level of need of the patient*, eligible patients receiving a "low-intensity" or "medium-intensity" Intervention, allocated according to the Algorithm depicted in Table 2.

**Table 2:** Algorithm for patient allocation to Intervention

DT^1 ^score <4		DT^1 ^score 4 or greater		
No risk factors	+ risk factors^2^ HADS^3 ^< 8	+/- risk factors^2^ HADS^3 ^< 8	+/- risk factors^2^ HADS^3 ^8-21	+/- risk factors^2^ HADS^3 ^22 or greater

Usual care	**Low-intensity Intervention** Patient self-directed resource suite only		**Medium-** **intensity** **Intervention** Tailored psychosocial Intervention delivered by trained Health Professional	Specialised Treatment with an appropriately qualified practitioner via a pre-defined clinical pathway

DT^1^: Distress Thermometer

Risk Factors^2^: Risk factors as identified in the National Breast and Ovarian Cancer Centre Psychosocial Checklist (PSCL)

HADS^3^: Hospital Anxiety and Depression Scale

#### i) Low-intensity Intervention

Patients who have a Hospital Anxiety and Depression Scale (HADS) [30] score of less than 8, *in addition *to *either *the presence of risk factors or current distress (DT score 4 or greater) will receive the low-intensity Intervention. This consists of a suite of consumer resources demonstrated to be effective and acceptable, including the consumer version of the Clinical Practice Guidelines for the Psychosocial Care of Adults with Cancer: *Cancer, how are you travelling? *[31]. The resource suite will include practical information about support groups and describe structured problem-solving and simple cognitive strategies, which can be employed to deal with concerns. Engagement of patients through educational and other resources has been demonstrated to be effective in collaborative care models [32] with the potential for patients to develop self-care and other strategies sustained beyond the duration of the Intervention. Engagement of patients in self-care strategies is strongly endorsed by consumer advisors to this study. In addition it has inherent appeal in terms of cost and potential to be delivered in diverse settings.

#### ii) Medium-intensity Intervention

Patients who have a HADS score of 8-21 inclusive will receive the medium-intensity Intervention, irrespective of the presence or absence of risk factors Simple provision of information alone has been demonstrated to be insufficient to treat depressed patients [33], meaning that a more intensive Intervention is required for depressed patients [34]. The medium-intensity Intervention consists of up to 4 tailored sessions, delivered by a health professional who has participated in specific training in order to be skilled to deliver the Intervention. These sessions will be conducted face-to-face or by telephone, depending on mutual convenience of patient and the health professional. All medium-intensity Intervention sessions will be up to 30 minutes in duration, conducted over a 4-week period. The duration of this Intervention reflects clinical practice with brief therapy, and represents a feasible time commitment for the health professional.

All medium-intensity Intervention sessions will have the following core principles:

• Focus on engagement with the patient

• Eliciting and exploring key patient concerns

• Establishment of an agreed treatment plan with the patient, focusing on strategies to address Distress. This treatment plan will be developed in consultation with the patient after review of their self-report on the Distress Thermometer as above. The treatment plan will incorporate detailed assessment and referral for further assistance as necessary, for example with practical concerns, financial issues, or physical symptoms such as pain or nausea. The need for referral will depend on the background training of the health professional delivering the Intervention - for example, a nurse will often be able to assess and initiate symptom management whilst a radiation therapist would refer the patient to a doctor or nurse.

• Regular review of this plan and its progress through the process of Clinical Case Review (clinical supervision).

The medium-intensity Intervention has been designed to ensure flexibility to select the most appropriate strategies to assist each patient, *based on the patient's self-reported areas of concern as identified on the Distress Thermometer*. During their training health professionals will acquire the necessary skills to critically analyse the nature of the patient's concerns and devise a tailored Intervention specific to the patient's needs. Examples of specific techniques which may be used by the health professional in the medium-intensity Intervention depending on patient need are provided in Table 3.

**Table 3:** Examples of strategies to address patient distress

Type of Distress	Specific techniques to address Distress
**Practical **- finances; difficulty with domestic tasks	Referral to Social Work; clarification of concerns; structured problem-solving; challenging black and white thinking about the need to perform domestic tasks; re-assigning priorities.

**Family **- concerns about children	Listening; acknowledgment of concerns; explanation about children's needs; discussion about the benefits of maintaining routine; reassuring children that they have not caused the cancer.

**Emotional **- anxiety about chemotherapy	Explanation; identification of automatic thoughts; challenging negative cognitions; relaxation and guided imagery.

**Physical - **pain	Referral for medical review; exploration of concerns about pain; identifying and challenging misbeliefs e.g. about becoming dependent on analgesia or that use of morphine implies inevitably poor prognosis; relaxation and guided imagery.

**Spiritual - **shame about dependence, low sense of worth	Dignity-conserving techniques e.g. exploration of past experiences, reflection on strengths; engaging in creative discussion about ways to feel in control; framing assistance as necessary to maintain dignity.

All health professionals delivering the medium-intensity Intervention will participate in Clinical Case Review (clinical supervision) facilitated by an experienced consultation-liaison psychiatrist. Sessions will be conducted in group format on a weekly basis. Health professionals will use these sessions to discuss the patients for whom they have delivered the medium-intensity Intervention, including discussion of specific components of the Intervention, difficulties encountered by the health professional, and be assisted to devise strategies to respond. A pre-determined structure will be followed to ensure consistency of Clinical Case Review across sites.

The health professional will record details of *each *session in a structured logbook noting precise details of all Intervention contacts with each patient including date, duration, mode (face-to-face or telephone), themes discussed and referrals such as to physiotherapy, or medical review of pain.

#### iii) High-intensity Intervention

Patients who have high levels of distress as measured on the HADS (scores of 22 or greater) have needs beyond the training of the health professionals participating in this study and require a high-intensity Intervention. All patients in this category will be referred for Specialised Treatment (high-intensity Intervention) with an appropriately qualified practitioner via a pre-defined clinical pathway at the respective site.

#### iv) Usual Care

Patients with *no *current distress (DT Score < 4) and *no *risk factors (on Psychosocial Checklist) will not receive any additional information or psychosocial support related to coping with cancer beyond the normal clinical practice at the site where they are receiving cancer care. They will continue to participate in follow-up or cancer-specific treatment as usual. They will not have contact with the trained health professionals at that site other than in the usual course of clinical care.

### Participants

#### Health professionals

Health professionals from the disciplines of nursing, occupational therapy, speech pathology, nutrition and dietetics, physiotherapy or radiation therapy will be eligible to participate in training if they: (1) have at least 12 months' clinical experience in oncology (2) are currently engaged in patient contact (minimum of 6 hours per week) (3) have a commitment to undertake the necessary training and (4) are working in a setting in which they can deliver the Intervention. Health professionals who meet the selection criteria will be offered entry into the study by the Investigators and will complete baseline measures before receiving a self-directed training manual and participating in a day-long skills development session. The core elements of the training will be (1) Supportive/expressive - encouraging expression of emotions; validating the individual's experiences; empathic listening, and provision of information (2) Cognitive-behavioural - teaching skills in problem-solving; identifying and challenging automatic thoughts; reframing; relaxation training and guided imagery (3) Dignity-conserving - encouraging reflection on themes of coherence, isolation, hope, helplessness, purpose and courage, as well as practical needs. This training will be completed over a period of 10 weeks. Psychologists, social workers and psychiatrists will not be eligible to participate as (1) it is considered that their core skills encompass the material included in the training, and (2) patients may be referred to these professionals as part of the intervention, for example a patient may be referred to a social worker because of financial concerns (as per Table 3). The number of health professionals to be trained at each site will vary depending on when that site is randomised. We aim to train sufficient numbers of health professionals so that no health professional is required to deliver the Intervention to more than 2 patients per Epoch (10-week cycle depicted in Table 1).

#### Patients

Inclusion criteria are: (1) aged 18 years or over (2) consecutive patients attending outpatient oncology clinics at each site (3) currently receiving active treatment for cancer OR have completed active treatment within the previous 2 months OR have been diagnosed with recurrent/advanced cancer within the previous 2 months. Exclusion criteria are: (1) inability to speak and read English, (2) receiving current specialised psychological treatment from a psychologist, psychiatrist, or other trained counsellor, (3) currently taking antidepressant medication, (4) health status precludes ability to complete questionnaires and participate in up to four psychosocial Intervention sessions and follow-up at 10 weeks, and (5) recruited in any previous Epoch of this study (that is, patients can only participate during one Epoch).

We will recruit 20 patients per site per Epoch (with the exception of the Training Epochs during which no patients will be recruited) with a total sample size of 600 patients. No patient who has been recruited in a previous Epoch will be eligible to participate in subsequent Epochs. Primary outcome measure is change in HADS score over ten weeks from enrolment to follow-up. Estimates of HADS in similar populations [35] are a mean of 17.8 with SD = 9.0, with a 10%-15% difference in mean change-scores assumed to be clinically significant. To detect this difference, with power = 80%, in a before-after design assuming a correlation 0.5 within patient over time, about 200 patients in each group are needed (Stata IC, version 10). Patients will be clustered by clinic, but we have no data on which to estimate intraclass correlation for the HADS scores; so will assume a design effect of deff = 1.5 to accommodate clustering, and hence we will require 600 patients in all; across five clinics, so we will seek to enrol 120 patients per site.

### Study Integrity

Ethical approval for this study has been obtained from the Human Research Ethics Committees of Royal Brisbane and Women's Hospital and the University of Queensland. The study design will be guided by the CONSORT statement [36]. Each site will be randomly allocated to the Training phase which will be immediately followed by Intervention phase, by the study statistician. Every consecutive patient attending the outpatient oncology department at each site will be screened for eligibility and enrolled if eligible and consenting. Careful documentation of outpatient attendance, subsequent enrolments and consent interviews will ensure that no patient is "selected" for inclusion or exclusion from the study. No patients will be randomised, and whether or not they receive the Intervention will be based on the algorithm depicted in Table 2. It is not possible to blind patients to the Intervention. No health professionals will be randomised. All participating health professionals will receive the training and deliver the psychosocial Intervention to eligible patients. It is not possible to blind health professionals delivering the psychosocial Intervention to the treatment status of patients.

### Measures

#### Health Professional assessment instruments

Demographic details, including professional background and training will be obtained at intake. The following measures will be completed at baseline and on completion of the study when the health professional has completed delivery of the psychosocial Intervention.

##### Psychological distress

The General Health Questionnaire 28 (GHQ) [37] will be used to assess general distress. This is a brief self-report measure which has been extensively used to screen for psychosocial morbidity, with good reliability and validity.

##### Stress and burnout

Occupational stress and burnout will be assessed with the Maslach Burnout Inventory (MBI) [38], a widely-used measure of occupational stress and burnout which assesses Emotional Exhaustion, Depersonalisation and Personal Accomplishment

##### Perceptions of participants

Semi-structured interviews will be conducted with a subset of five health professionals from each site to examine their perceptions of the Training and delivery of the Intervention, including practical aspects of delivery and impediments to the Intervention, as well as reflections about their experience of Clinical Case Review and perceived impact of participation in the study on their clinical practice.

#### Patient assessment instruments

Demographic details, disease stage and current treatment status will be obtained at intake. The following measures will be completed at baseline and 10-week follow-up.

##### Distress

The Distress Thermometer will be used to assess distress. The Distress Thermometer is a self-rated instrument in which patients indicate their Distress (0 = No distress; 10 = Extreme distress), nominating the source of Distress: Practical; Physical; Family; and Emotional or Spiritual/Religious [14]. A cut-off score of 4 has the greatest sensitivity and specificity compared with other validated measures [39].

##### Psychosocial Risk Factors

The Psychosocial Checklist is a structured template based on evidence in the Clinical Practice Guidelines for the Psychosocial Care of Adults with Cancer about known risk factors for development of depression and anxiety in cancer patients [15]. It has been successfully implemented in multiple clinical services across Australia.

##### Mood

The Hospital Anxiety and Depression Scale (HADS) [30] will be used to assess mood. This 14-item scale has good reliability and validity and has been extensively used in studies of cancer patients, cut-off scores of 22 and above representing severe disorder, and less than 8 representing no disorder.

##### Unmet Psychological Supportive Care Needs

The Supportive Care Needs Survey-Short-Form SCNS-SF 34 [40] will assess patients' needs for help over the last month across the following domains: Psychological; Health Systems and Information; Patient Care and Support, and Physical and Daily Living needs.

##### Quality of life

FACT-G (Functional Assessment of Cancer Scale - General) [41] is a widely-used measure of quality of life in the cancer population, with well-being subscales: Physical; Social/family; Emotional, and Functional.

EQ-5 D (EuroQoL) [42] is a brief self-report measure of quality of life from which a QALY can be calculated. This measure has been used in intervention studies with depressed patients [43].

##### Experience of demoralisation

DS (Demoralisation Scale) is a 24 item self-report scale with a Cronbach alpha of .96, measuring depression commonly seen in the physically ill [44]. Norms have been established in a non-clinical sample [45].

##### Health service use

Patients will maintain a record of visits to General Practitioners and any contact with community or hospital-based support services, using a structured template.

### Statistical analyes

#### Quantitative analysis

We will compute and compare the average change in HADS scores among patients enrolling during Intervention Epochs with scores of patients enrolling during Control Epochs. The strength of the stepped-wedge design is that we will be able to account for systematic differences between sites and times during the trial, and also for case-mix differences between patients.

We will use a patient-level hierarchical analysis model to accommodate these potentially confounding factors. With this model, we can adjust for case-mix differences between sites, as well as allow for specific site characteristics (other than presence/absence of the Intervention). A generalised estimating equation (GEE) linear or logistic model will be implemented [28] to estimate the effect of the Intervention on the patients' change in HADS scores and the prevalence of depression "caseness" at each site. Stata Version 10 (StataCorp, 2008) will be used for all statistical analyses.

#### Qualitative analysis

Semi-structured interviews with health professionals will be tape-recorded, transcribed verbatim and analysed to determine similarity of themes or differences between barriers to delivery of supportive care previously identified [46]. Clinical Case Review sessions will be recorded, transcribed and similarly analysed.

A random selection of 10% of all HP log books will be examined to determine adherence to the training and identify patterns of referral (for example because of pain) and delineate professional, patient or system barriers to delivery of the Intervention.

## Discussion

This study will examine the effectiveness and feasibility of a model of psychosocial care in which patients are systematically screened for psychosocial needs and risk and then assigned to receive therapy tailored to their precise level of need. Use of novel and readily accessible service providers to deliver the Intervention after participation in focused training is an innovative approach designed to improve patient access to timely psychosocial care, and represents an efficient approach in the face of limited access to scarce psychosocial resources. Inclusion of patients who are considered at risk of anxiety and depression even if not currently distressed is an important strategy to examine the potential to prevent the development of more serious distress. The brief flexible mode of delivery as part of cancer clinicians' routine practice is aimed to address the stigma of psychosocial care and facilitate uptake by patients whose physical condition would make attendance for longer therapies difficult. Incorporation of clinical supervision is a central platform to assist health professionals delivering the Intervention, ensure quality of care, monitor patient responses and reduce the potential for development of health professional stress. The incorporation of in-depth interviews with health professional participants and detailed analysis of the process of clinical supervision will provide fine-grained insights into enablers and barriers of this model of care and guide refinement before its more widespread implementation. Recognition of emotional distress as a vital clinical sign in cancer care has been a major achievement in cancer care policy. One of the barriers to achieving the intended goals of such policy in psychosocial care is the effective translation of the substantial body of evidence regarding screening and psychosocial treatments into routine clinical practice by a range of cancer clinicians in a range of settings. This study aims to help address this gap in knowledge, by training health professionals who work closely with cancer patients in daily practice. More importantly an innovative component of this study is the support and mentoring of health professionals providing psychosocial care as a strategy to bridge knowledge and practice change, while using mixed methodology to study the processes of such clinical practice change.

## Competing interests

DC is a member of the Research Advisory Group of *beyondblue*, the National Depression Initiative. He absented himself from reviews of grant applications and was not involved in discussion of any funding decisions.

## Authors' contributions

JT, BK and DC developed the study concept, aims and initiated the project. PY, SA, DJ, SC, MH and LMcF assisted further in the refinement of the concept and development of the protocol. JT, BK, DC, SA and MH will implement the protocol and oversee collection of data. JT, BK and DC will provide health professional training and clinical supervision, in consultation with other authors. JT was responsible for the drafting of the manuscript. All authors have read and approved the final manuscript.

## Pre-publication history

The pre-publication history for this paper can be accessed here:

http://www.biomedcentral.com/1471-2407/11/48/prepub
